# Renin-angiotensin system blockers and residual kidney function loss in patients initiating peritoneal dialysis: an observational cohort study

**DOI:** 10.1186/s12882-017-0616-4

**Published:** 2017-06-17

**Authors:** Jenny I. Shen, Anjali B. Saxena, Sitaram Vangala, Satvinder K. Dhaliwal, Wolfgang C. Winkelmayer

**Affiliations:** 1 Division of Nephrology and Hypertension, Los Angeles Biomedical Research Institute at Harbor-UCLA Medical Center, 1124 W. Carson St., C-1 Annex, Torrance, CA 90502 USA; 2 Division of Nephrology, Department of Medicine, Stanford University School of Medicine, Palo Alto, CA USA; 3 Department of Medicine Statistics Core, David Geffen School of Medicine at UCLA, Los Angeles, CA USA; 4 Selzman Institute for Kidney Health, Section of Nephrology, Department of Medicine, Baylor College of Medicine, Houston, TX USA

**Keywords:** Peritoneal dialysis, Renin angiotensin system blockers, Angiotensin converting enzyme inhibitors, Angiotensin receptor blockers, Residual kidney function

## Abstract

**Background:**

Although angiotensin-converting enzyme inhibitors (ACEI) and angiotensin-II receptor blockers (ARB) have been shown to preserve residual kidney function in a select group of Asian patients undergoing continuous ambulatory peritoneal dialysis (PD) in two small randomized clinical trials, the effectiveness of these drugs has yet to be demonstrated in a more diverse population of patients with multiple comorbid conditions. We investigated the association between ACEI/ARB use and development of recorded anuria in a cohort of patients initiating PD in the U.S.

**Methods:**

We conducted a retrospective observational cohort study using the US Renal Data System and electronic health records data from a large national dialysis provider. We identified adult patients who initiated PD from 2007 to 2011. Only patients who participated in the federal prescription drug benefit program, Medicare Part D, for the first 90 days of dialysis were included. Patients who filled a prescription for an ACEI or ARB during those 90 days were considered users. We applied Cox proportional hazards models to an inverse probability of treatment-weighted (IPTW) cohort to estimate the hazard ratio (HR) for anuria (24-h urine volume < 200 ml) in ACEI/ARB users vs. non-users.

**Results:**

Among 886 patients, 389 (44%) used an ACEI/ARB. Almost a third of these patients were black or Hispanic, and more than a quarter had comorbidities that would have excluded them from the randomized clinical trials of ACEI/ARB. Two hundred eighty patients reached anuria over 840 person-years of follow-up, for a composite event rate of 33 events per 100 person-years. We found no clear association between ACEI/ARB use and progression to anuria [HR: 0.86, 95% CI: 0.73–1.02].

**Conclusions:**

ACEI/ARB use is common in patients initiating PD in the U.S. but was not associated with a lower risk of anuria. Residual confounding by unmeasured variables is an important limitation of this observational study. Still, these findings suggest that pragmatic clinical trials are warranted to test the effectiveness of ACEI/ARB in slowing the decline of residual kidney function in a diverse population of peritoneal dialysis patients with multiple comorbid conditions.

**Electronic supplementary material:**

The online version of this article (doi:10.1186/s12882-017-0616-4) contains supplementary material, which is available to authorized users.

## Background

Preservation of residual kidney function has been consistently associated with improved outcomes in patients with end-stage kidney disease undergoing peritoneal dialysis (PD). A reanalysis of the Canada-USA Peritoneal Dialysis Cohort showed that for every 5 L/week per 1.73 m^2^ increase in residual kidney function, the risk of death decreased by 12% [1]. Similar results were obtained from the ADEquacy of PD in MEXico (ADEMEX) and NEtherlands COoperative Study on the Adequacy of Dialysis (NECOSAD) studies [2, 3]. Residual kidney function has also been linked to improved volume and phosphorus control, less anemia, improved nutrition, and decreased inflammation [4–9].

One of the few promising interventions to slow the decline of residual kidney function has been the use of angiotensin-converting enzyme inhibitors (ACEI) and angiotensin-II receptor blockers (ARB). Two small randomized trials demonstrated a slower rate of decline in residual kidney function in those treated with the ACEI, ramipril, or the ARB, valsartan, vs. placebo [10, 11]. But these trials had several limitations: both were very small and included only Asian patients on continuous ambulatory PD, excluding those on cyclers, the PD modality used by over 50% of U.S. PD patients [12]. They also excluded patients with comorbidities common in PD patients, including heart failure, recent myocardial infarction, stroke, valvular disease, and chronic liver disease. Thus, their findings may have limited generalizability to the general PD population in the U.S. Further complicating the picture is a large cohort study of incident Dutch PD patients that, in contrast to the randomized trials, showed no benefit associated with ACEI or ARB use [13].

In this retrospective observational cohort study, we examined whether ACEI or ARB use was associated with preservation of residual kidney function in a large, ethnically and racially diverse cohort of U.S. patients initiating PD from 2007 to 2011.

## Methods

### Study population

**Fig. 1:**
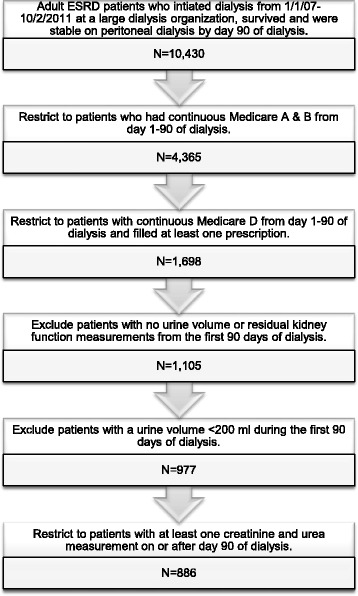
Study population selection from the United States Renal Data System. We selected a cohort of adult patients initiating peritoneal dialysis between 2007 and 2011 in the U.S. with DaVita, Inc. who survived to day 90 of dialysis with residual renal function (24-h urine volume ≥ 200 ml), and who had continuous Medicare Parts A, B, and D coverage from day 1 to 90. ESRD – end-stage renal disease

From the United States Renal Data System (USRDS), we retrospectively identified all adult (≥18 years old) patients with ESRD who initiated dialysis between January 1, 2007 and October 2, 2011 (Fig. 1). We restricted the cohort to patients who survived and were stable on PD (i.e., on the modality for at least 60 days) by day 90 of dialysis, the index date. Thus, index dates ranged from April 1, 2007 to December 31, 2011. Inclusion criteria included continuous Medicare Parts A, B, and D coverage (elements of a federal health insurance program for people who are 65 or older, certain younger people with disabilities, and people with end-stage kidney disease) from day 1 to 90 of dialysis and having had at least one prescription filled during that time as an indication of active participation in the prescription drug benefit program (Medicare Part D). All patients had to be dialyzing with DaVita, Inc., a large dialysis organization. Patients were excluded if they were reported as anuric (24-h urine volume < 200 ml) during the first 90 days of dialysis or had no residual kidney function measurements after day 90.

### ACEI/ARB use

Use of ACEI/ARB (versus no use) was the exposure of interest and defined using Medicare Part D insurance claims for prescription drugs. Patients were categorized as ACEI/ARB users if they filled a prescription for either ACEI or ARB within 90 days of initiating dialysis; everyone else was considered a non-user. For analyses using an approach that corresponds to an “intention-to-treat” analysis in trials, baseline exposure was carried forward indefinitely. “As-treated” analyses considered patients exposed for 60 days after the recorded supply from their previously filled prescription was exhausted (“refill grace period”). If patients failed to fill a subsequent prescription during this 60-day grace period, the follow-up time was censored. Conversely, follow-up for non-users was censored if an ACEI/ARB prescription was filled.

### Residual kidney function

All laboratory measurements were obtained from the electronic health record of DaVita, Inc. For the repeated measures analysis, residual kidney function, or residual glomerular filtration rate (rGFR), was calculated as the mean of 24-h creatinine and urea clearances corrected for body surface area (ml/min/1.73 m^2^). For the survival analysis, anuria was defined as urine volume < 200 ml/24 h.

### Patient characteristics

We ascertained demographics [age, sex, race (white, black, other), Hispanic ethnicity, Medicaid (a federal health insurance program for low-income patients) at time of dialysis initiation], comorbidities, body mass index (BMI), baseline medication use, dialysis characteristics (year initiated dialysis, pre-dialysis referral to nephrologist), and facility characteristics (size of the PD program, rural/urban location, U.S. census division) from the Medical Evidence Report (form CMS-2728), the ESRD Facility Survey (form CMS-2744) conducted in the year a patient initiated dialysis, and all available Medicare claims data from the first 90 days of dialysis. Details about these algorithms have been previously described and can be found in Additional file 1: Table S1 [14]**.** Laboratory measurements (hemoglobin, albumin, baseline rGFR), were obtained from DaVita Inc.

### Statistical analysis

We tabulated the characteristics of ACEI/ARB users and non-users using percentages and means (+/− standard deviations) or medians (interquartile range) and compared the two groups using standardized differences. As per convention, we considered variables with a standardized difference > 0.1 to be imbalanced between the groups [15].

Two measures of loss of residual kidney function, 1) the rate of rGFR decline and 2) time to anuria, were evaluated for association with ACEI/ARB use. To analyze the first measure, we used spaghetti plots and locally weighted smoothing (LOESS) to visually compare the change in rGFR over time between the users and non-users. We then used a linear mixed effects model including drug use, time, and their interaction as fixed effects, and a patient random effect to account for repeated measurements. Users and non-users were assumed to have equal mean rGFR at baseline. Patients were censored when they transferred to and remained on hemodialysis for >60 days. In essence, this model compares the slope of decline of rGFR between users and non-users adjusting for the fact that each patient had several urine volume measurements over time. We performed the analyses on an inverse probability of treatment weighted (IPTW) cohort to control for selection bias for observed characteristics between ACEI/ARB users and non-users [16]. The method first involves generating a propensity score (PS), which is the predicted probability of being treated with an ACEI/ARB adjusting for the variables listed in Table 1, with the exception of BMI as these data were not available for all patients. Note that we achieved balance in the IPTW cohort for BMI even though it was excluded from the propensity score modeling. Users were then weighted by the inverse of their probability of being treated with an ACEI/ARB (i.e. 1/PS) and non-users by the inverse of their probability of not being treated [i.e. 1/(1-PS)] to create a pseudo-population in which ACEI/ARB users and non-users have a similar distribution of characteristics, simulating the balance ideally achieved in a randomized study, albeit only for the variables included in the propensity score modeling. Please see the In-Depth Methods in the Additional file 1 for detailed information on this method.

**Table 1:** Characteristics of U.S. patients initiating peritoneal dialysis from 2007 to 2011 with Medicare Part D coverage

	Full cohort			IPTW Cohort		
Variable	Non-users *N* = 497	ACEI/ARB users *N* = 389	Std. diff.	Non-users	ACEI/ARB users	Std. diff.
Demographics						
Age (yr, mean ± SD)	67 ± 14	64 ± 13	0.22	66 ± 19	65 ± 19	0.05
Male sex	56	56	0.00	56	57	0.02
Race						
Black	16	17	0.03	16	16	0.00
White	79	76	0.07	78	77	0.02
Other	5	7	0.08	6	7	0.04
Hispanic ethnicity	10	16	0.18	12	12	0.00
Medicaid at time of dialysis initiation	26	34	0.18	28	28	0.00
Reported comorbidities						
Cancer	11	6	0.18	9	8	0.04
Cardiac disease, other^a^	21	20	0.02	20	22	0.05
Cerebrovascular disease	9	11	0.07	9	9	0.00
Coronary artery disease	21	23	0.05	21	22	0.02
Diabetes mellitus	55	64	0.18	60	58	0.04
Heart failure	29	25	0.09	28	28	0.00
Hyperkalemia	3	3	0.00	3	3	0.00
Hyperlipidemia	18	18	0.00	18	18	0.00
Hypertension	92	96	0.17	93	94	0.04
Liver disease	2	1	0.08	1	2	0.08
Peripheral vascular disease	15	16	0.03	15	15	0.00
Pulmonary disease	15	13	0.06	14	14	0.00
Tobacco use	7	7	0.00	7	7	0.00
Days hospitalized in in the first 90 days of dialysis (median, IQR)	0 (0–0)	0 (0–0)	0.00	0 (0–0)	0 (0–0)	0.00
Baseline medication use						
ACEI or ARB						
ACEI	0	65	NA	0	65	NA
ARB	0	42	NA	0	41	NA
Both	0	6	NA	0	6	NA
Beta blocker	63	61	0.04	63	62	0.02
Calcium channel blocker	46	64	0.37	54	57	0.06
Diuretic	56	66	0.21	61	59	0.04
Other antihypertensive^b^	40	48	0.16	43	44	0.02
Statin	49	61	0.24	54	56	0.04
Clopidogrel	12	13	0.03	13	14	0.03
Warfarin	10	6	0.15	9	8	0.04
Other cardiovascular med^c^	19	25	0.15	21	23	0.05
Levothyroxine	17	18	0.03	18	18	0.00
Dialysis characteristics						
Saw nephrologist prior to dialysis initiation	88	88	0.00	87	89	0.06
Year initiated dialysis						
2007	14	18	0.11	15	16	0.03
2008	17	21	0.10	18	21	0.08
2009	15	18	0.08	17	16	0.03
2010	29	25	0.09	28	27	0.02
2011	25	18	0.17	22	20	0.05
CAPD (vs. CCPD)	32	40	0.17	35	36	0.02
Vital signs and laboratory measurements						
BMI (mean ± SD)^d^	28.3 ± 6	29.0 ± 6.4	0.11	28.4 ± 8	28.8 ± 9.7	0.04
Hemoglobin (g/dL, mean ± SD)	10.7 ± 1.4	10.9 ± 1.5	0.14	10.7 ± 1.9	10.9 ± 2.2	0.10
Albumin (g/dL, mean ± SD)	3.8 ± 0.5	3.8 ± 0.5	0.00	3.8 ± 0.6	3.8 ± 0.8	0.00
Baseline rGFR (ml/min, mean ± SD)	8.4 ± 4.8	8.5 ± 4.7	0.02	8.3 ± 6.3	8.6 ± 7.2	0.07
24 h urine volume (ml, median, IQR)	900 (550–1400)	1000 (600–1500)	0.07	850 (600–1400)	900 (600–1400)	0.01
Facility characteristics						
Number of PD patients (median, IQR)^e^	24 (14–39)	25 (14–39)		24 (14–39)	25 (14–39)	
≥20	62	65	0.06	62	62	0.00
Rural^f^	14	15	0.03	15	15	0.00
Geographic location (U.S. census division)^g^						
East North Central	12	13	0.03	13	12	0.03
East South Central	7	8	0.04	8	7	0.04
Middle Atlantic	8	6	0.08	7	8	0.04
Mountain	5	3	0.10	4	5	0.05
New England	5	3	0.10	5	3	0.10
Pacific	10	16	0.18	13	14	0.03
South Atlantic	29	27	0.04	28	27	0.02
West North Central	10	8	0.07	10	10	0.00
West South Central	13	15	0.06	13	14	0.03

All numbers are percentages unless indicated otherwise. *ACEI* angiotensin-converting enzyme inhibitor, *ARB* angiotensin-II receptor blocker, *BMI* body mass index, *CAPD* continuous ambulatory peritoneal dialysis, *CCPD* continuous cycling peritoneal dialysis, *eGFR* estimated glomerular filtration rate, *IQR* interquartile range, *IPTW* inverse probability of treatment weighted, *PD* peritoneal dialysis, *SD* standard deviation, *Std. Diff*. standardized difference

^a^Includes atrial fibrillation, arrhythmias, implanted cardiac defibrillators, pacemakers, and valvular disease

^b^Includes alfuzosin, aliskiren, clonidine, doxazosin, guanfacine, hydralazine, isosorbide, methyldopa, minoxidil, prazosin, ranolazine, and terazosin

^c^Includes ezetimibe, simvastatin, niacin, amiodarone, aspirin/dipyridamole, colesevelam, colestipol, digoxin, dipyridamole, dronedarone, fenofibrate, flecainide, gemfibrozil, mexiletine, nitroglycerin, omega-3 acid ethyl esters, procainamide, propafenone, and quinidine

^d^Missing for 11% of non-users and 1% of users

^e^Based on the year the patient initiated dialysis

^f^Facilities were considered urban if they were classified as a metropolitan area in the Rural–Urban Commuting Area (RUCA) Codes version 2.0, which are based on 2000 Census commuting data and 2004 zip codes; all other areas were considered to be rural [24]

^g^Facilities were categorized into one of nine U.S. Census Bureau Divisions based on their state [25]

To study the association between ACEI/ARB use and the development of anuria, our second measure of residual kidney function decline, we used Cox proportional hazard models to compare time to developing anuria between users and non-users. Patients were censored for death, kidney transplantation, switch to hemodialysis for >60 days, loss to follow-up in the DaVita, Inc., system, and end of study (January 1, 2012). For as-treated analyses, patients were additionally censored when they crossed over to the other group: ACEI/ARB users were censored when they were assumed to be out of medications and thus no longer using the drug (60 days past when their most recent recorded prescription expired), and non-users were censored when they filled an ACEI/ARB prescription, thereby becoming ACEI/ARB users. We performed the analyses on an inverse probability of treatment weighted (IPTW) cohort to control for selection bias for observed characteristics between ACEI/ARB users and non-users [16]. All hazard ratios (HR) were accompanied by their corresponding 95% confidence interval (CI). We assessed effect modification by age (< or ≥66 years, the mean age of our cohort), sex, race, history of diabetes mellitus, history of coronary artery disease, history of heart failure, and PD modality (continuous ambulatory peritoneal dialysis vs. continuous cycling peritoneal dialysis).

We examined the robustness of our primary results against potential outliers in a sensitivity analysis in which we restricted the cohort to patients whose baseline rGFR was ≤20 ml/min to ensure that outliers with high rGFR were not driving the results. To test whether short follow-up times may have biased the results, we also ran sensitivity analyses restricting the cohort to patients who were still alive, on PD, and making urine a year after starting dialysis. Follow-up time was calculated from day 365 of dialysis (for the primary analysis the index date was day 90 of dialysis).

All analyses were performed using SAS 9.4 (SAS Institute Inc., Cary, NC).

## Results

### Patient characteristics

We identified 1698 adult patients who initiated PD from 2007 to 2011 and had continuous Medicare parts A, B, and D from day 1–90 of dialysis. Of these, 812 were excluded for either lack of residual kidney function measurements or a urine volume < 200 ml during the first 90 days of dialysis. The remaining 886 patients were included in the cohort. The excluded patients had similar demographic characteristics to those in the analytic cohort (Table 1 and Additional file 1: Table S2). Notably 42% of excluded patients were ACEI/ARB users; this is comparable to the 44% of included patients who were ACEI/ARB users.

The overall cohort of included patients was comprised of 16% black patients and 13% Hispanic patients. ACEI/ARB users were younger and more likely to be Hispanic and receiving Medicaid (Table 1). Although there was no difference in the baseline prevalence of coronary artery disease or heart failure between the two groups, diabetes mellitus and hypertension were more common among users, while cancer was more prevalent among non-users. Users had higher rates of anti-hypertensive and statin use, but a lower rate of warfarin use. However, their use of other medications was comparable to those of non-ACEI/ARB users. On average, ACEI/ARB users had higher BMI and hemoglobin than non-users, but comparable rGFR and 24-h urine volume at baseline. A smaller percentage of ACEI/ARB users started dialysis in 2011 compared to non-users, and a higher percentage of them used continuous ambulatory peritoneal dialysis (CAPD) rather than continuous cycling peritoneal dialysis (CCPD). The two groups had similar facility characteristics. After weighting the cohort by their inverse probability of treatment with ACEI/ARB, all observed characteristics were balanced between users and non-users (Table 1) [17].

### Association of ACEI/ARB use with rate of loss of rGFR

**Fig. 2:**
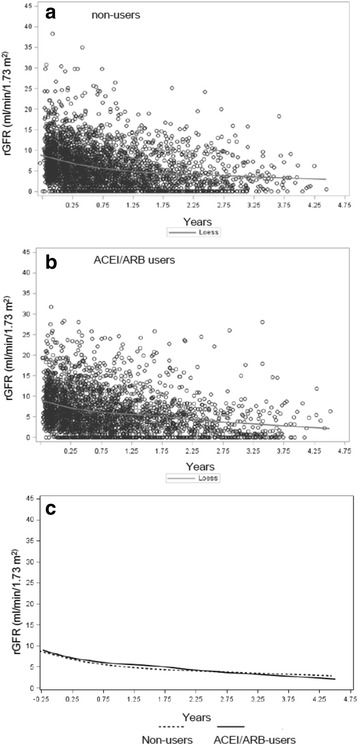
Locally weighted smoothing (LOESS) curves of the decline in residual glomerular filtration rate (rGFR) over time in Panel (**a**) non-users, (**b**) angiotensin-converting enzyme inhibitors (ACEI) and angiotensin-II receptor blockers (ARB) users, and Panel (**c**) user and non-user curves superimposed

At baseline, ACEI/ARB users and non-users had nearly identical mean rGFR of 8.5 and 8.4 ml/min/1.73 m^2^. On average, patients’ rGFR declined by 1.8 ml/min per year. When we used locally weighted smoothing (LOESS) and spaghetti plots to visually compare the slope of decline in rGFR, we did not appreciate any difference between the two groups over time (Fig. 2, Additional file 1: Figure S1). We were unable to obtain consistent results when modeling the data using linear mixed effect models so were unable to formally test for a difference in the loss of rGFR.

### Association of ACEI/ARB use with time to Anuria

In the intention-to-treat analysis, 280 patients reached anuria over 840 person-years of follow-up, for a composite event rate of 33 events per 100 person-years (Table 2). This rate was no different between ACEI/ARB users and non-users (HR 0.86, 95% CI: 0.73–1.02). Age (≥66 years old vs. <66 years old), sex, race, history of diabetes mellitus, history of coronary artery disease, history of heart failure, and PD modality did not modify any of the associations. The as-treated analysis did detect an association of ACEI/ARB use with a nearly 40% reduction in the risk of anuria compared to non-users (Table 2), but was limited by a very short median follow-up time of 2.6 months.

**Table 2:** Number of patients, events, follow-up time, incidence rates, and hazard ratios for anuria in an IPTW cohort

Cohort	Analysis	Exposure group	N	Number of events	Follow-up time (years)		Incidence rate (per 100 person-years)	Hazard ratio (95% CI)
					Mean ± SD	Median		
Full cohort	ITT	ACEI/ARB	389	121	1.05 ± 0.89	0.81	29.6	0.86 (0.73, 1.02)
		Non-user	497	159	0.87 ± 0.82	0.62	36.9	
Full cohort	AT	ACEI/ARB	389	53	0.35 ± 0.37	0.24		0.66 (0.51, 0.84)
		Non-user	497	91	0.30 ± 0.33	0.19		
Baseline rGFR ≤20 ml/min	ITT	ACEI/ARB	379	118	1.05 ± 0.89	0.80		0.87 (0.74, 1.03)
		Non-user	485	156	0.88 ± 0.82	0.62		
≥1 year on PD cohort	ITT	ACEI/ARB	203	59	0.93 ± 0.80^a^	0.71^a^		0.98 (0.76, 1.27)
		Non-user	209	58	0.85 ± 0.78^a^	0.63^a^		

*ACEI* angiotensin-converting enzyme inhibitor, *ARB* angiotensin-II receptor blocker, *AT* as treated, *CI* confidence interval, *IPTW* inverse probability of treatment weighted, *ITT* intention to treat, *rGFR* residual glomerular filtration rate, *SD* standard deviation

^a^Note that follow-up for the ≥1 year on PD cohort began on day 365 of dialysis whereas in the other analyses follow-up began on day 90 of dialysis. Thus, patients in the ≥1 year on PD cohort were followed on average until day 690 of dialysis whereas patients in the unadjusted ITT analysis of the full cohort were followed on average until day 436 of dialysis

To ensure that the results were not driven by outliers with high rGFR, we performed sensitivity analyses restricted to those whose rGFR was ≤20 ml/min. The point estimates for the hazard ratios were similar to that of the main analyses, though the results did not reach statistical significance (Additional file 1: Table S3, Table 2).

To test whether short follow-up times may have biased the results of the primary analysis, we also ran a second set of sensitivity analyses restricting the cohort to patients who were on PD for at least a year. The point estimate was close to one (HR 0.98, 95% CI: 0.76–1.27) with wider confidence intervals (Table 2).

Anuria may have been under-ascertained if patients stopped collecting their urine when they neared or reached anuria. So, we calculated for each subject the interval between the last urine collection and the end up of follow-up. Since urine collections are generally done every 90 days, we considered those whose last collection interval was >100 days to be “late collectors”. We found that ACEI/ARB users had a higher proportion of “late collectors” than non-users (6% vs. 3% *p* = 0.02), suggesting that anuria was more likely to be under-ascertained in ACEI/ARB users rather than non-users.

## Discussion

In this study’s intention-to-treat analyses, ACEI and ARB use was not associated with a reduction in the risk of anuria in a large, diverse cohort of patients initiating PD in the U.S. We also did not appreciate a difference in the slope of decline of rGFR between the groups. While ACEI/ARB use was associated with a reduction in the risk of anuria in as-treated analyses, the applicability of these findings is limited since the median follow-up time in those analyses was less than 3 months. Overall, our findings suggest that ACEI/ARB use may not preserve residual kidney function in the general population of U.S. patients initiating PD, a population in which this intervention had previously not been well-studied.

Our null findings are in contrast to two small randomized controlled studies examining the effect of ACEI or ARB on residual kidney function in patients on PD: one randomized 60 prevalent patients to 5 mg of ramipril daily vs. placebo; the other randomized 34 incident patients to 40–80 mg of valsartan daily vs. placebo [10, 11]. Both showed a slower rate of decline in rGFR in those treated vs. placebo. However, the studies had several limitations, most notably the limited generalizability of the results. The trials were small and only included Asian patients living in Hong Kong or Japan using continuous ambulatory PD. By comparison, less than 7% of U.S. patients initiating PD are Asian, and more than half use a cycler [12]. Furthermore, the trials excluded patients with conditions common to patients on PD, including congestive heart failure, myocardial infarction within the last 6 months, clinically significant valvular disease, malignant hypertension, history of hypertensive encephalopathy or cerebrovascular accident within the last 6 months, chronic liver disease, malignant disease, and known history of bilateral renal artery stenosis. These criteria would render at least a quarter of our study cohort ineligible. Thus, one possible explanation for the discrepancy between the findings of our study versus the two trials is that these drugs may not be effective in preserving residual kidney function in a more racially and ethnically diverse population with a high burden of comorbid conditions.

A limitation of this study is that ACEI and ARB may have been started for indications other than to preserve residual kidney function, such as for improved cardiovascular outcomes after an acute myocardial infarction or for heart failure. However, these indications support lifelong use of the drugs, if tolerated, at dosages equivalent to or higher than those used in the trials for residual kidney function. Thus, if ACEI and ARB are effective in preserving residual renal function in patients with such cardiovascular conditions, we would have expected to see a benefit to ACEI and ARB use in our cohort regardless of the indication for the medication.

Of note, a meta-analysis of four studies comparing ACEI or ARB with other antihypertensive drugs for preservation of residual kidney function in patients using PD included the two previously mentioned trials as well as two published in Chinese [18]. It found that long-term (≥12 months), but not short-term, use of ACEI or ARB was effective in patients using continuous ambulatory PD. The median follow-up time in our study was only about 8–9 months, which might further explain why we found no significant association in the primary analyses. However, when we restricted the cohort to patients who remained on PD for at least a year, ACEI or ARB use was still not significantly associated with a reduction in the risk for anuria. More importantly, the hazard ratios in these sensitivity analyses were quite close to one, suggesting that even a well-powered analysis would have only found an arguably clinically insignificant 2–3% reduction in the risk of anuria.

Our findings are consistent with those of a recent observational cohort study of 452 patients initiating PD from 1997 to 2007 in the Netherlands [13]. As in our study, the NECOSAD cohort included patients with cardiovascular disease, cerebrovascular disease, and congestive heart failure. Investigators found that ACEI or ARB use was not associated with either a change in the decline in rGFR (*p* = 0.52) or with a decreased risk of developing anuria (HR: 0.95, 95% CI: 0.60–1.44), and results were consistent in both intention-to-treat and as-treated analyses. A smaller observational cohort study of 156 patients who initiated PD in Australia from 1995 to 2001 similarly found no benefit to ACEI/ARB use (HR 0.81, 95% CI: 0.52–1.27) [19]. By contrast, the largest observational study to date followed 1032 patients initiating PD in the U.S. in 1996 and 1997 and detected a beneficial association between ACEI use and the risk of anuria (odds ratio 0.70, *p*-value 0.02) [20]. Of note, the distribution of CAPD vs. CCPD in that era is the reverse of what it is now: 64% of PD patients used CAPD in 1997, while 64% of our cohort used a cycler instead [12]. Also, although the investigators controlled for baseline estimated GFR, they did not adjust for baseline urine volume, an important confounder since the outcome of anuria was defined as 24-h urine volume < 200 ml, and there was not a strong correlation between rGFR and urine volume.

While we did not find that using ACEI or ARB was associated with an attenuation in the decline of residual kidney function in the general population of U.S. patients initiating PD, these medications may still have an important role in the treatment of patients on PD. As shown in the randomized clinical trials, they may be effective in preserving rGFR in patients on CAPD, in certain ethnic groups, or in a select group of patients with no major comorbidities. There is also evidence that these classes of medications may reduce the risk of cardiovascular events in patients on PD [21, 22]. However, pragmatic clinical trials are needed to test the effectiveness of ACEI and ARB in slowing the decline of rGFR in patients seen in routine clinical practice, a diverse population that often has other medical diseases.

Our study has limitations. Most significantly, we could not control for unmeasured confounders, including blood pressure and the specific indication for the drug, specific dosage, and prior duration of its use. It is possible that patients treated with an ACEI or ARB had poorer blood pressure control, which can accelerate the loss of residual kidney function, and that this outweighed any potential benefit of the drug itself. Furthermore, we did not have data on patient’s ultrafiltration, volume, or membrane transport status, use of biocompatible dialysis solutions, proteinuria, nor their history of peritonitis or other acute illnesses, all of which can affect urine volume. Imbalance in these unmeasured variables could account for our null findings. We also did not restrict our cohort to new users of ACEI or ARB, although studying prevalent users tends to overestimate the beneficial effect of a drug since these patients have tolerated and adhered to the medication and thus tend to be healthier than those who may have discontinued the drug shortly after initiation [23]. We also may have under-ascertained anuria events in subjects who stopped collecting urine once they neared or reached anuria. However, since such “late collectors” were more likely to be ACEI/ARB users, this also would have biased our results towards a beneficial association between ACEI/ARB use and anuria. These limitations must be balanced against the strengths of the study, which include a large, racially and ethnically diverse incident cohort of patients on PD with a high burden of comorbidities, a group that is usually excluded from clinical trials, and the use of IPTW to minimize indication bias.

## Conclusion

In conclusion, we did not find ACEI or ARB use to be associated with a decreased risk of anuria in a large, diverse cohort of patients with multiple comorbidities initiating PD in the U.S. from 2007 to 2011, although the observational study was limited by residual confounding. While there is a high level of evidence supporting the use these medications in preserving residual kidney function in a select group of patients, further pragmatic clinical trials are needed to test the effectiveness of these drugs in the general PD population.

## Additional file

Additional file 1: Table S1. Comorbidity definition by USRDS Medical Evidence Report comorbid condition and ICD9 diagnosis code. Comorbidities were assigned either if they were present on the Medical Evidence Report, or if coded in at least one inpatient claim or two outpatient claims at least 1 day apart. **Table S2.** Characteristics of U.S. patients initiating peritoneal dialysis from 2007 to 2011 excluded from the analysis for missing residual kidney function data. **Table S3.** Characteristics of patients initiating peritoneal dialysis from 2007 to 2011 whose baseline residual glomerular filtration rate was ≤20 ml/min. **Figure S1.** Spaghetti plots of residual glomerular filtration rate in Panel A) non-users, and in Panel B) angiotensin-converting enzyme inhibitors (ACEI) and angiotensin-II receptor blockers (ARB) users. In-Depth Methods. Inverse Probability of Treatment Weighting Approach (PDF 805 kb)

## Abbreviations

ACEI: Angiotensin-converting enzyme inhibitors
ADEMEX: ADEquacy of PD in MEXico
ARB: Angiotensin-II receptor blockers
BMI: Body mass index
CAPD: Continuous ambulatory peritoneal dialysis
CCPD: Continuous cycling peritoneal dialysis
CI: Confidence interval
HR: Hazard ratio
IPTW: Inverse-probability of treatment-weighted
LOESS: Locally weighted smoothing
NECOSAD: NEtherlands COoperative Study on the Adequacy of Dialysis
PD: Peritoneal dialysis
rGFR: Residual glomerular filtration rate
USRDS: United States Renal Data System
