# Supplementary material for: Renin-angiotensin system blockers and residual kidney function loss in patients initiating peritoneal dialysis: an observational cohort study
Source: BMC Nephrol. 2017 Jun 17;18:196. doi: 10.1186/s12882-017-0616-4 (PMC5473971; doi:10.1186/s12882-017-0616-4)
Supplement: Additional file 1: Table S1. — Comorbidity definition by USRDS Medical Evidence Report comorbid condition and ICD9 diagnosis code. Comorbidities were assigned either if they were present on the Medical Evidence Report, or if coded in at least one inpatient claim or two outpatient claims at least 1 day apart. Table S2. Characteristics of U.S. patients initiating peritoneal dialysis from 2007 to 2011 excluded from the analysis for missing residual kidney function data. Table S3. Characteristics of patients initiating peritoneal dialysis from 2007 to 2011 whose baseline residual glomerular filtration rate was ≤20 ml/min. Figure S1. Spaghetti plots of residual glomerular filtration rate in Panel A) non-users, and in Panel B) angiotensin-converting enzyme inhibitors (ACEI) and angiotensin-II receptor blockers (ARB) users. In-Depth Methods. Inverse Probability of Treatment Weighting Approach (PDF 805 kb) [file 12882_2017_616_MOESM1_ESM.pdf]

Table S1: Comorbidity definition by USRDS Medical Evidence Report comorbid condition and ICD9 diagnosis code[1].

Comorbidity	Medical Evidence Report comorbid condition	ICD9 diagnosis code
Cancer	Malignant neoplasm, cancer	140.x-165.x, 170.x-172.x, 174.x, 175.x, 180.x-209.x, 238.6, 273.3
Cardiac disease, other		427.3x, 426.x, 427.0, 427.20, 427.81, 427.9, 37.9x, 996.04, v53.32, 37.7x, 37.8x, 996.01, v45.00, v45.01, v45.02, v45.09, v53.31, 394.x-397.1, 424.x, 746.3-746.6, v42.2, v43.3, 427.1, 427.4x
Cerebrovascular disease	Cerebrovascular disease cerebrovascular accident, transient ischemic attack	433.x, 434.x, 437.x, 438x, 433.x, 434.x, 436.00, 435.x
Coronary artery disease	Atherosclerotic heart disease	410.x, 412, 414.0x, 36.2x, 36.1x, 00.66, 36.06, 36.07, 36.09, 411.x, 413.x
Diabetes mellitus	Diabetes, currently on insulin<br>Diabetes, on oral medications<br>Diabetes, without medications<br>Diabetic retinopathy	249.x, 250.x, 357.2, 362.0x
Heart failure	Congestive heart failure	398.91, 402.01, 402.11, 402.91, 404.01, 404.03, 404.13, 404.91, 428.x
Hyperkalemia		276.7
Hyperlipidemia		272.x
Hypertension		401.0, 401.1, 401.9, 402.x, 403.x, 404.x, 405.x
Liver disease	NA	070.x, 456.1, 456.21, 570-573.x, v42.7
Peripheral vascular disease	Peripheral vascular disease<br>Amputation	38.03, 38.04, 38.05, 38.08, 38.33-38.48, 39.22-39.29, 440.2x, 440.3x, 440.4x, 441.x, 443.x, 445.x, 447.10, 557.10, 557.90, v43.4
Pulmonary disease	Chronic obstructive pulmonary disease	490, 491.x-496, 500-505, 506.4, 516.x, 416.x, 417.9
Tobacco use		305.1x

[1] Comorbidities were assigned either if they were present on the Medical Evidence Report, or if coded in at least one inpatient claim or two outpatient claims at least 1 day apart.

Table S2: Characteristics of U.S. patients initiating peritoneal dialysis from 2007-2011 excluded from the analysis for missing residual kidney function data.

Variable	Full cohort		
	**Non-users N=473**	**ACEI/ARB users N=339**	**Std. diff.**
**Demographics**			
Age (yr, mean ± SD)	67 ± 13	65 ± 13	0.13
Male sex	51	43	0.14
Race			
Black	20	23	0.06
White	74	72	0.06
Other	5	5	0.00
Hispanic ethnicity	9	18	0.26
Medicaid at time of dialysis initiation	27	38	0.23
**Reported comorbidities**			
Cancer	13	9	0.14
Cardiac disease, other [a]	30	24	0.14
Cerebrovascular disease	13	15	0.06
Coronary artery disease	27	23	0.11
Diabetes mellitus	58	73	0.30
Heart failure	32	31	0.02
Hyperkalemia	4	7	0.12
Hyperlipidemia	22	23	0.04
Hypertension	92	97	0.24
Liver disease	3	3	0.00
Peripheral vascular disease	16	19	0.07
Pulmonary disease	17	16	0.04
Tobacco use	6	6	0.02
Days hospitalized in in the first 90 days of dialysis (median, IQR)	0 (0-4)	0 (0-5)	0.04
**Baseline medication use**			
ACEI or ARB			
ACEI	0	60	NA
ARB	0	47	NA
Both	0	7	NA
Beta blocker	58	65	0.15
Calcium channel blocker	54	60	0.13
Diuretic	53	70	0.36
Other antihypertensive [b]	38	43	0.09
Statin	49	53	0.08
Clopidogrel	15	14	0.04
Warfarin	9	7	0.05

Other cardiovascular med[c]	21	21	0.00
Levothyroxine	18	17	0.04
**Dialysis characteristics**			
Saw nephrologist prior to dialysis initiation	82	82	0.00
Year initiated dialysis			
2007	19	20	0.04
2008	20	20	0.01
2009	20	22	0.05
2010	21	20	0.04
2011	21	19	0.05
CAPD (vs. CCPD)	44	50	0.13
**Vital signs and laboratory measurements**			
BMI (mean ± SD) [d]	28.8 ± 7.2	29.6 ± 6.7	0.11
Hemoglobin (g/dL, mean ± SD)	10.5 ± 1.7	10.6 ± 1.4	0.07
Albumin (g/dL, mean ± SD)	3.5 ± 0.6	3.5 ± 0.7	0.08
**Facility characteristics**			
Number of PD patients (median, IQR) [e]	26 (15-41)	24 (14-43)	0.03
≥20	64	62	0.03
Urban [f]	89	88	0.03
Geographic location (U.S. census division) [g]			
East North Central	16	17	0.02
East South Central	9	8	0.03
Middle Atlantic	7	4	0.13
Mountain	5	4	0.04
New England	6	7	0.03
Pacific	13	21	0.20
South Atlantic	18	19	0.03
West North Central	9	4	0.20
West South Central	17	17	0.01

All numbers are percentages unless indicated otherwise. ACEI angiotensin-converting enzyme inhibitor; ARB angiotensin-II receptor blocker; BMI body mass index; CAPD continuous ambulatory peritoneal dialysis; CCPD continuous cycling peritoneal dialysis; eGFR estimated glomerular filtration rate; IQR interquartile range; IPTW inverse probability of treatment weighted; PD peritoneal dialysis; SD standard deviation; Std. Diff. standardized difference.

[a] Includes atrial fibrillation, arrhythmias, implanted cardiac defibrillators, pacemakers, and valvular disease.

[b] Includes alfuzosin, aliskiren, clonidine, doxazosin, guanfacine, hydralazine, isosorbide, methyldopa, minoxidil, prazosin, ranolazine, and terazosin.

[c] Includes ezetimibe, simvastatin, niacin, amiodarone, aspirin/dipyridamole, colesevelam, colestipol, digoxin, dipyridamole, dronedarone, fenofibrate, flecainide, gemfibrozil, mexiletine, nitroglycerin, omega-3 acid ethyl esters, procainamide, propafenone, and quinidine.
[d] Missing for 11% of non-users and 1% of users.
[e] Based on the year the patient initiated dialysis.
[f] Facilities were considered urban if they were classified as a metropolitan area in the Rural–Urban Commuting Area (RUCA) Codes version 2.0, which are based on 2000 Census commuting data and 2004 zip codes; all other areas were considered to be rural.[1]
[g] Facilities were categorized into one of nine U.S. Census Bureau Divisions based on their state.[2]

Table S3: Characteristics of patients initiating peritoneal dialysis from 2007-2011 whose baseline rGFR was ≤ 20 ml/min.

Variable	Full cohort			IPTW Cohort		
	**Non-users N=485**	**ACEI/ARB users N=379**	**Std. diff.**	**Non-users**	**ACEI/ARB users**	**Std. diff.**
**Demographics**						
Age (yr, mean ± SD)	66 ± 14	64 ± 13	0.15	66 ± 19	66 ± 19	0.00
Male sex	44	43	0.02	56	57	0.02
Race						
Black	16	17	0.03	16	16	0.00
White	79	75	0.10	78	78	0.00
Other	5	7	0.08	6	6	0.00
Hispanic ethnicity	10	15	0.15	11	12	0.03
Medicaid at time of dialysis initiation	27	34	0.15	28	28	0.00
**Reported comorbidities**						
Cardiac disease, other [a]	21	20	0.02	20	20	0.00
Cerebrovascular disease	9	11	0.07	9	9	0.00
Coronary artery disease	21	23	0.05	22	22	0.00
Diabetes mellitus	54	64	0.20	59	58	0.02
Heart failure	29	25	0.09	27	27	0.00
Hyperkalemia	3	3	0.00	3	3	0.00
Hyperlipidemia	18	17	0.03	17	17	0.00
Hypertension	92	96	0.17	94	94	0.00
Liver disease	2	1	0.08	1	1	0.00
Peripheral vascular disease	15	16	0.03	15	15	0.00
Pulmonary disease	15	13	0.06	14	14	0.00
Tobacco use	7	7	0.00	7	7	0.00
Days hospitalized in in the first 90 days of dialysis (median, IQR)	0 (0-0)	0 (0-0)	0.11	0 (0-0)	0 (0-0)	0.02
**Baseline medication use**						
ACEI or ARB						
ACEI	0	58	NA	0	59	NA
ARB	0	35	NA	0	36	NA
Both	0	7	NA	0	6	NA
Beta blocker	63	62	0.02	63	62	0.02
Calcium channel blocker	47	64	0.35	55	56	0.02
Diuretic	56	66	0.21	60	60	0.00
Other antihypertensive [b]	40	49	0.18	43	43	0.00
Statin	50	61	0.22	55	56	0.02
Clopidogrel	12	13	0.03	13	13	0.00
Warfarin	10	7	0.11	8	8	0.00
Other cardiovascular med[c]	19	25	0.15	21	22	0.02
**Dialysis characteristics**						
Saw nephrologist prior to	88	88	0.00	89	89	0.00

dialysis initiation						
Year initiated dialysis						
2007	15	19	0.11	16	17	0.03
2008	17	21	0.10	19	20	0.03
2009	15	17	0.05	16	16	0.00
2010	29	25	0.09	27	27	0.00
2011	25	18	0.17	22	21	0.02
CAPD (vs. CCPD)	32	40	0.17	36	37	0.02
**Vital signs and laboratory measurements**						
BMI (mean ± SD) [d]	28.3 ± 5.8	29.0 ± 6.4	0.12	28.4 ± 7.9	28.8 ± 9.7	0.05
Hemoglobin (g/dL, mean ± SD)	10.7 ± 1.5	10.9 ± 1.5	0.13	10.8 ± 2.0	10.8 ± 2.2	0.00
Albumin (g/dL, mean ± SD)	3.8 ± 0.5	3.8 ± 0.5	0.00	3.8 ± 0.6	3.8 ± 0.7	0.00
Baseline rGFR (ml/min, mean ± SD)	7.9 ± 4.0	8.1 ± 4.0	0.05	7.9 ± 5.3	8.2 ± 6.0	0.05
24 hour urine volume (ml, median, IQR)	850 (550-1350)	923 (550-1500)	0.07	850 (550-1300)	900 (600-1400)	0.03
**Facility characteristics**						
Number of PD patients (median, IQR) [e]	24 (14-39)	26 (14-40)	0.13	24 (15-39)	25 (14-39)	0.05
≥20	62	65	0.06	63	62	0.02
Rural [f]	14	15	0.03	15	15	0.00
Geographic location (U.S. census division) [g]						
East North Central	12	13	0.03	13	13	0.00
East South Central	7	7	0.00	8	8	0.00
Middle Atlantic	8	6	0.08	6	6	0.00
Mountain	4	3	0.05	4	4	0.00
New England	5	3	0.10	4	4	0.00
Pacific	11	16	0.15	13	13	0.00
South Atlantic	29	27	0.04	28	28	0.00
West North Central	11	8	0.10	10	10	0.00
West South Central	13	15	0.06	14	14	0.00

All numbers are percentages unless indicated otherwise. ACEI angiotensin-converting enzyme inhibitor; ARB angiotensin-II receptor blocker; BMI body mass index; CAPD continuous ambulatory peritoneal dialysis; CCPD continuous cycling peritoneal dialysis; rGFR residual glomerular filtration rate; IQR interquartile range; IPTW inverse probability of treatment weighted; PD peritoneal dialysis; SD standard deviation; Std. Diff. standardized difference.

[a] Includes atrial fibrillation, arrhythmias, implanted cardiac defibrillators, pacemakers, and valvular disease.
[b] Includes alfuzosin, aliskiren, clonidine, doxazosin, guanfacine, hydralazine, isosorbide, methyldopa, minoxidil, prazosin, ranolazine, and terazosin.
[c] Includes ezetimibe, simvastatin, niacin, amiodarone, aspirin/dipyridamole, colesevelam, colestipol, digoxin, dipyridamole, dronedarone, fenofibrate, flecainide, gemfibrozil, mexiletine, nitroglycerin, omega-3 acid ethyl esters, procainamide, propafenone, and quinidine.
[d] Missing for 2% of non-users and 1% of users.

[e] Based on the year the patient initiated dialysis.
[f] Facilities were considered urban if they were classified as a metropolitan area in the Rural–Urban Commuting Area (RUCA) Codes version 2.0, which are based on 2000 Census commuting data and 2004 zip codes; all other areas were considered to be rural.[1]
[g] Facilities were categorized into one of nine U.S. Census Bureau Divisions based on their state.[2]

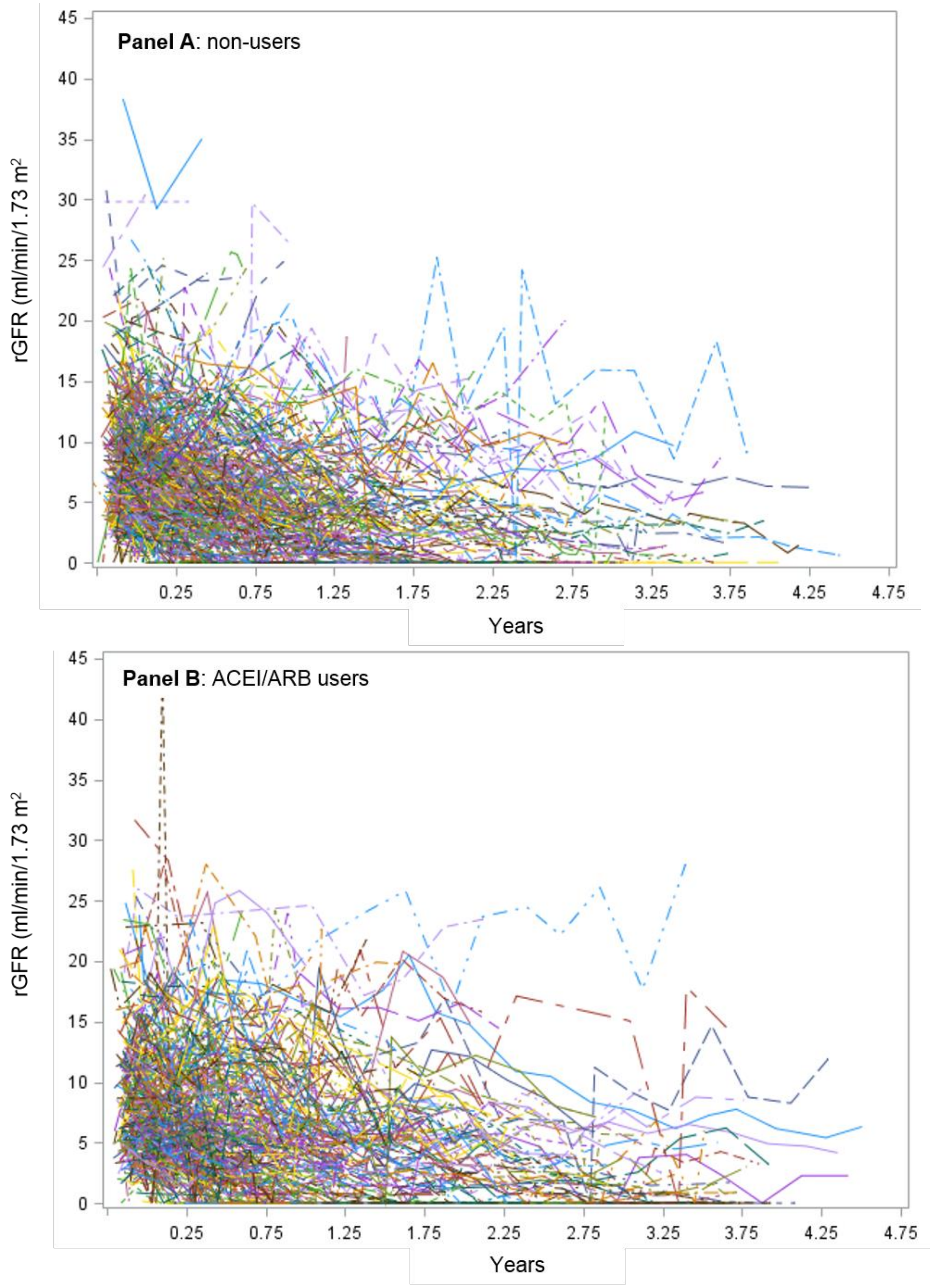

Figure S1: Spaghetti plots of residual glomerular filtration rate in Panel **A**) non-users, and in Panel **B**) angiotensin-converting enzyme inhibitors (ACEI) and angiotensin-II receptor blockers (ARB) users.

In-Depth Methods: Inverse Probability of Treatment Weighting Approach

The use of the weights is a method to simulate what would have happened had everyone in the population experienced both levels of exposure.[3] A simple application of the weights creates a pseudo-population of double size but this double size does not imply higher precision because each individual is used twice. By applying stabilization (multiplying the weight by the probability of being exposed for those exposed and the probability of being unexposed for those unexposed), we are able to create a pseudo-population with similar percentage of patients exposed in each level of the covariates as the overall percentage in the study population. As explained in Hernan and Robins 2006, inverse probability of treatment weighting (IPTW) mimics a design I randomized experiment that eliminates selection bias by observed characteristics between warfarin users and non-users and with use of stabilized weights the pseudo-population has the same size as the original population.

Implementation The following steps show how to implement IPTW:

Step 1. Run a multivariate logistic regression model to predict the probability of a patient being exposed ($\hat{p}_{Ti}$), adjusted for all variables listed in Table 1 with the exception of body mass index.

Step 2. Compute a weight for each patient in each exposure group. For a patient *i* in the exposed group with N_T patients, $w_i = \frac{1}{\hat{p}_i}$, For a patient *j* in the unexposed group with N_C patients, $w_j = \frac{1}{1-\hat{p}_j}$

Step 3. Compute the Stabilized Weights (sw).[4,5] To compute stabilized weights one multiplies the weight found in Step 2 above by the probability of being exposed (p_T) for those exposed and the probability of being unexposed for those unexposed $(1 - p_T)$. More specifically the stabilized weight, sw, is defined as: for a patient *i* in the exposed group, $sw_i = p_T * w_i$ and for a patient j in the unexposed group, $sw_j = (1 - p_T) * w_i$, In our cohort, this would imply multiplying by 0.44 for ACE/ARB users and 0.56 for ACE/ARB non-users which are the percentages of patients in the treated and not treated groups, respectively.

Step 4. If necessary, truncate weights to the value 10 (0.1) if stabilized weights are found to be too large (small).[6] As a check and also as a tool to decide on a model specification, the estimated weights are required to have a mean of 1 (approximately).[5]

Analysis Comparison of statistics (mean, proportions) between exposed and unexposed in the pseudo-population can be done through the use of procedures (functions) that allow the use of weights. Similarly standardized differences were computed with the use of the weighted means, weighted proportions and weighted standard deviations. We used weighted robust Cox proportional hazards models to obtain parameter estimates and SE.

References

1. WWAMI Ruca Rural Health Research Center. http://depts.washington.edu/uwruca/ruca-data.php. Accessed January 5, 2012, 2012.
2. U.S. Census Bureau GD. Census Bureau Regions and Divisions with State FIPS Codes. http://www.census.gov/geo/www/reg_div.txt. Accessed January 5, 2012, 2012.
3. Hernan MA, Robins JM. Estimating causal effects from epidemiological data. J Epidemiol Community Health. 2006;60(7):578-586.
4. Robins JM, Hernan MA, Brumback B. Marginal structural models and causal inference in epidemiology. Epidemiology. 2000;11(5):550-560.
5. Cole SR, Hernan MA. Constructing inverse probability weights for marginal structural models. Am J Epidemiol. 2008;168(6):656-664.
6. Harder VS, Stuart EA, Anthony JC. Propensity score techniques and the assessment of measured covariate balance to test causal associations in psychological research. Psychol Methods. 2010;15(3):234-249.

STROBE Statement—Checklist of items that should be included in reports of ***cohort studies***

	Item No		Recommendation
**Title and abstract**	1	✓	(*a*) Indicate the study's design with a commonly used term in the title or the abstract
		✓	(*b*) Provide in the abstract an informative and balanced summary of what was done and what was found
			**Introduction**
Background/rationale	2	✓	Explain the scientific background and rationale for the investigation being reported
Objectives	3	✓	State specific objectives, including any prespecified hypotheses
			**Methods**
Study design	4	✓	Present key elements of study design early in the paper
Setting	5	✓	Describe the setting, locations, and relevant dates, including periods of recruitment, exposure, follow-up, and data collection
Participants	6	✓	(*a*) Give the eligibility criteria, and the sources and methods of selection of participants. Describe methods of follow-up
		N/A	(*b*) For matched studies, give matching criteria and number of exposed and unexposed
Variables	7	✓	Clearly define all outcomes, exposures, predictors, potential confounders, and effect modifiers. Give diagnostic criteria, if applicable
Data sources/ measurement	8*	✓	For each variable of interest, give sources of data and details of methods of assessment (measurement). Describe comparability of assessment methods if there is more than one group
Bias	9	✓	Describe any efforts to address potential sources of bias
Study size	10	✓	Explain how the study size was arrived at
Quantitative variables	11	✓	Explain how quantitative variables were handled in the analyses. If applicable, describe which groupings were chosen and why
Statistical methods	12	✓	(*a*) Describe all statistical methods, including those used to control for confounding
		✓	(*b*) Describe any methods used to examine subgroups and interactions
		✓	(*c*) Explain how missing data were addressed
		✓	(*d*) If applicable, explain how loss to follow-up was addressed
		✓	(*e*) Describe any sensitivity analyses
			**Results**
Participants	13*	✓	(a) Report numbers of individuals at each stage of study—eg numbers potentially eligible, examined for eligibility, confirmed eligible, included in the study, completing follow-up, and analysed
		✓	(b) Give reasons for non-participation at each stage
		✓	(c) Consider use of a flow diagram
Descriptive data	14*	✓	(a) Give characteristics of study participants (eg demographic, clinical, social) and information on exposures and potential confounders
		✓	(b) Indicate number of participants with missing data for each variable of interest
		✓	(c) Summarise follow-up time (eg, average and total amount)
Outcome data	15*	✓	Report numbers of outcome events or summary measures over time
Main results	16	✓	(*a*) Give unadjusted estimates and, if applicable, confounder-adjusted estimates and their precision (eg, 95% confidence interval). Make clear which

			confounders were adjusted for and why they were included
		✓	(*b*) Report category boundaries when continuous variables were categorized
		✓	(*c*) If relevant, consider translating estimates of relative risk into absolute risk for a meaningful time period
Other analyses	17	✓	Report other analyses done—eg analyses of subgroups and interactions, and sensitivity analyses
			**Discussion**
Key results	18	✓	Summarise key results with reference to study objectives
Limitations	19	✓	Discuss limitations of the study, taking into account sources of potential bias or imprecision. Discuss both direction and magnitude of any potential bias
Interpretation	20	✓	Give a cautious overall interpretation of results considering objectives, limitations, multiplicity of analyses, results from similar studies, and other relevant evidence
Generalisability	21	✓	Discuss the generalisability (external validity) of the study results
			**Other information**
Funding	22	✓	Give the source of funding and the role of the funders for the present study and, if applicable, for the original study on which the present article is based

*Give information separately for exposed and unexposed groups.

Note: An Explanation and Elaboration article discusses each checklist item and gives methodological background and published examples of transparent reporting. The STROBE checklist is best used in conjunction with this article (freely available on the Web sites of PLoS Medicine at http://www.plosmedicine.org/, Annals of Internal Medicine at http://www.annals.org/, and Epidemiology at http://www.epidem.com/). Information on the STROBE Initiative is available at http://www.strobe-statement.org.
